# Review on Aging Behavior and Durability Enhancement of Bamboo Fiber-Reinforced Polymer Composites

**DOI:** 10.3390/molecules30153062

**Published:** 2025-07-22

**Authors:** Sameeksha Shettigar, Mandya Channegowda Gowrishankar, Manjunath Shettar

**Affiliations:** Department of Mechanical and Industrial Engineering, Manipal Institute of Technology, Manipal Academy of Higher Education, Manipal 576104, India

**Keywords:** bamboo fiber composites, aging behavior, durability, water absorption, fiber treatment, hybrid composites, environmental degradation

## Abstract

This review article focuses on the long-term durability challenges associated with bamboo fiber-reinforced polymer composites when subjected to various environmental aging conditions such as water immersion, hygrothermal fluctuations, ultraviolet (UV) radiation, soil burial, and refrigerated storage. The primary issue addressed is the degradation of mechanical and structural performance of bamboo fiber-reinforced polymer composites due to moisture absorption, fiber swelling, and fiber–matrix interface deterioration. To mitigate these aging effects, the study evaluates and compares multiple strategies, including chemical and physical fiber surface treatments, filler additions, and fiber hybridization, which aim to enhance moisture resistance and mechanical stability. These composites are relevant in automotive interiors, construction panels, building insulation, and consumer goods due to their eco-friendly nature and potential to replace conventional synthetic composites. This review is necessary to consolidate current knowledge, identify effective enhancement approaches, and guide the development of environmentally resilient bamboo fiber-reinforced polymer composites for real-world applications.

## 1. Introduction

Natural fiber composites (NFCs) are newly found, green alternatives to traditional synthetic fiber-reinforced composites [1]. NFCs incorporate fibers from renewable plant sources like flax [2], jute [3], hemp [4], sisal [5], coir [6], and bamboo [7] into polymer matrices. Natural fibers offer many benefits, such as being biodegradable, renewable, readily available, and cheaper than synthetic materials like glass or carbon fibers [8]. Most significantly, they contribute much less to the environment. Due to these advantages, NFCs are becoming increasingly popular in the automotive, construction, and consumer products sectors, particularly where environmental impact and material weight reduction are highly preferred. Due to their low density, they are particularly suitable for producing lightweight materials, and their strength-to-weight ratio provides good mechanical performance. They also possess good heat and sound insulation properties and are safe to touch, being non-toxic and non-abrasive. All these features make NFCs an appealing choice for applications that do not need high performance but value cost-effectiveness and sustainability.

Bamboo fiber has become increasingly well-known as a green reinforcement for polymer composites in the last ten years due to its rare mix of good mechanical properties, eco-friendliness, and affordability [9,10]. Bamboo, one of the world’s fastest-growing plants, may be harvested in three to five years, which is significantly more renewable than conventional wood or most other natural fibers. It grows quickly with little pesticide or fertilizer requirement, making it a cost-effective and sustainable raw material even more attractive. Bamboo fiber possesses high strength in tension, good stiffness, and a remarkable strength-to-weight ratio, hence ideal for both structural and semi-structural applications [11,12]. These mechanical properties are primarily owed to its elongated cellulose microfibrils and relatively high lignin content, which give strength and stiffness and resist biodegradation. With all these benefits, bamboo-reinforced composites have the potential to be a viable alternative for synthetic fiber composites in diverse applications with similar performance but with much reduced environmental impact.

Besides its good mechanical properties, bamboo fiber also possesses high thermal and sound insulation properties, rendering it highly applicable for construction and car interiors. Its high silica content in nature provides fire-retardant capability when applied as a composite material. It also has the benefit of biodegradability, which can minimize the footprint at the end of its life and cater to the principles of a circular economy [13]. Bamboo’s charm has also increased through its common occurrence in Asia, Africa, and Latin America, where it has been utilized in traditional construction and manufacturing [14]. Using locally sourced bamboo in these regions helps the local economies and reduces transportation emissions associated with transporting products long distances due to its proximity to manufacturers’ facilities [15].

Understanding bamboo fiber-reinforced polymer composites’ durability and aging behavior is essential to assess their long-term performance in actual applications. While natural fiber composites provide obvious environmental advantages as well as mechanical benefits, they can be prone to degradation under environmental stress. Moisture exposure, variations in temperature, UV radiation, and chemical exposure can progressively alter the structure and functionality of these materials [16]. Over time, such conditions may lead to fiber swelling, matrix cracking, fiber–matrix debonding, and noticeable declines in key mechanical properties like strength, stiffness, and impact resistance [17].

Composites are often exposed to long-term environmental loading when used in automobile interiors, architectural materials, and patio furniture. The material might prematurely fail if aging is poorly understood, resulting in safety issues, increased maintenance expenses, and reduced service life. Thus, such a durability evaluation is vital for improving the material’s performance and the aim of user safety and product sustainability. Moreover, understanding aging mechanisms enables better design of longer-lasting composite materials by selecting fiber treatments, matrix modifications, and protective coatings. It also allows for predictive modeling of performance and lifespan, based on industrial applications and standards. For the case of bamboo fiber composites, which are more hydrophilic in nature than man-made ones, testing the extent to which aging affects moisture absorption, dimensional stability, and interfacial adhesion is particularly pertinent [18].

Bamboo fiber composites’ long-term sustainability hinges on their durability. Resistance to environmental aging is essential in determining their potential for structural and semi-structural applications. With industries trending towards environmentally friendly materials, knowing how these composites age is vital in ensuring reliability and facilitating wider acceptance.

Therefore, the primary objective of this review is to critically evaluate the aging behavior of bamboo fiber-reinforced polymer composites under diverse environmental conditions, such as water immersion, hygrothermal aging, ultraviolet radiation, soil burial, and freeze–thaw cycling. This study further aims to examine various durability enhancement strategies, including fiber surface treatments, filler incorporation, and hybridization techniques, that mitigate the adverse effects of aging. By synthesizing current research findings, the review provides guidance for developing more robust and sustainable bamboo fiber-reinforced polymer composites suitable for engineering applications in construction, automotive, and consumer sectors.

## 2. Bamboo Fiber Polymer Composites

Bamboo fiber is obtained from bamboo plant culms or stems and is a family of natural lignocellulosic fibers. Its special composition renders it a good option for strengthening polymer composites. Bamboo is generally around 50–60% cellulose, 20–25% hemicellulose, 15–20% lignin, and traces of waxes, silica, and ash [19]. The high cellulose content contributes the most to its remarkable tensile strength and stiffness, while lignin contributes to the structural stiffness of the fiber and enhances thermal resistance. While these ratios can differ depending on elements such as the bamboo species, age, growing environment, and fiber processing method, they give bamboo its exceptional mechanical performance when used as a composite material [20].

Another aspect differentiating bamboo fiber is its tubular and hierarchical microstructure, which enhances its strength-to-weight ratio and renders it relatively light in weight. The morphology also bestows good thermal insulation and sound-damping properties. Nonetheless, its surface is hydrophilic and rough due to the cellulose and hemicellulose hydroxyl groups.

### 2.1. Processing of Bamboo Fibers

Processing bamboo fibers is a crucial step that determines the structural strength, surface characteristics, and overall performance of the fibers in composite materials. There are many extraction and processing methods for bamboo fibers, including mechanical, chemical, and biological processes, which provide different advantages and limitations depending on the intended application and environmental considerations [21].

Mechanical processing [22] is the physical separation of the fibers from the bamboo culms through processes like crushing, rolling, and decortication. This method maintains the natural structure of the fibers and does not generate chemical waste; hence, it is more eco-friendly. The final fibers, though, can include residual lignin, hemicellulose, and pith material that can influence uniformity and minimize interfacial bonding in composites. Chemical treatment [23] generally uses alkalis (sodium hydroxide), acids, or bleaches to dissolve non-cellulosic contents and leave behind cellulose-enriched fibers. Such a process generates cleaner, finer fibers with improved consistency and polymer matrix adhesion. Alkali treatment is especially effective in dissolving hemicellulose and lignin, raising surface roughness, activating more hydroxyl groups, and improving the interaction between fiber and matrix. Despite these benefits, chemical processing is environmentally demanding because it involves the use of toxic chemicals and the requirement for effective effluent treatment. Biological processing (or enzymatic retting) [24] is a new green technology that employs enzymes or microbial activity to break down the binding agents in bamboo, allowing for fiber separation. While this process is slower and still being researched for optimization, it provides a cleaner alternative with a little environmental footprint.

Following extraction, the fibers are usually subjected to surface treatments for enhanced compatibility with hydrophobic polymer matrices. The commonly employed treatments are alkali (NaOH), silane, acetylation, or coupling agents that decrease hydrophilicity, improve thermal stability, and enhance mechanical interlocking at the fiber–matrix interface. The different types of bamboo fiber treatments, viz., chemical, physical, and biological, are presented and discussed by Ahmad et al. [25] in their article.

### 2.2. Fabrication of Bamboo Fiber-Reinforced Polymer Composites

Various methods are employed in fabricating bamboo fiber-reinforced polymer composites, with the choice of method depending on several key factors. First, methods that enable control over the alignment of fibers are beneficial for improving mechanical characteristics such as strength and rigidity. Second, the process must accommodate the intended fiber loading within the composite material. Third, the selection should correspond to the specific demands of the end-use application, be it in the automotive sector, building industry, or consumer products. Lastly, the thermal stability of both the bamboo fibers and the polymer matrix must be considered in determining the most suitable method. Hot pressing, injection molding, compression molding, and hand lay-up are frequently used fabrication techniques. The various methods for fabricating bamboo fiber-reinforced polymer composites are shown in Table 1.

## 3. Water Absorption Behavior of Bamboo Fiber-Reinforced Polymer Composites

Water absorption in bamboo fiber composites occurs through several mechanisms. The most common pathway is diffusion, where water molecules migrate through the polymer matrix, especially if it is semi-permeable or has low cross-linking density [43]. Capillary action through microcracks, voids, or fiber pull-outs created during composite fabrication also provides a route for water ingress [44]. Additionally, water can penetrate the fiber–matrix interface if there is poor adhesion between the two, a frequent issue in untreated natural fiber composites [45]. Upon absorbing water, bamboo fibers swell, which increases internal stress and can lead to debonding at the fiber–matrix interface. This process further accelerates water uptake, creating a self-reinforcing cycle of degradation.

The extent and rate of water absorption depend on several factors. One crucial factor is fiber content: increasing the volume fraction of bamboo fibers generally leads to greater water absorption due to increased hydrophilic sites and interfacial regions [46]. Another influential factor is fiber surface treatment. Untreated bamboo fibers are highly moisture-sensitive [47].

Bamboo fiber composites typically exhibit Fickian diffusion behavior over time during the initial stages of water absorption [48]. In this phase, water uptake increases proportionally with time, eventually reaching an equilibrium state called saturation. However, in prolonged exposure, the behavior can deviate into non-Fickian or anomalous diffusion [49] due to factors like matrix plasticization, fiber swelling, microcrack formation, or degradation at the fiber–matrix interface. Table 2 presents water uptake (%) by different bamboo fiber-reinforced polymer composites.

### 3.1. Remedies to Minimize the Water Absorption of Bamboo Fiber-Reinforced Polymer Composites

Many remedies, viz., surface treatments of fibers, adding filler materials, hybridization, and so on, have been discussed to minimize the water absorption of bamboo fiber-reinforced polymer composites. The following paragraphs contain a full discussion of various remedies.

#### 3.1.1. Surface Treatments of Fibers

Surface treatments are a crucial strategy for reducing the water absorption behavior of bamboo fiber-reinforced polymer composites. Bamboo fibers are inherently hydrophilic due to the high hydroxyl (-OH) group content in cellulose, hemicellulose, and lignin [56]. When exposed to humid environments, these polar groups form hydrogen bonds with water molecules, making the fibers prone to moisture absorption. This causes the fibers to swell and degrade the fiber–matrix interface, leading to microcracking, dimensional instability, and a reduction in mechanical properties over time. Surface treatments aim to mitigate these issues by chemically modifying or physically altering the fiber surface, thereby improving the composites’ moisture resistance and overall durability [57,58].

In the work by Zhuo et al. [55] bamboo fibers are treated with three different enzymes. Compared to untreated bamboo fiber–poly(hydroxybutyrate-co-valerate) composites, the treated fiber composites’ water absorption decreases by 6% to 26% under 10 days of distilled water-soaking conditions. Nabinejad et al. [59] has investigated the moisture sorption behavior of bamboo fiber polyester composites subjected to alkali (NaOH) surface treatment. The study reveals that untreated bamboo fiber composites exhibited the highest water sorption rates (12%), nearly twice as high as those of treated counterparts (6%). This behavior is mainly due to the abundant presence of lignin and hemicellulose on the surface of untreated fibers, both of which contain hydroxyl groups that exhibit a strong affinity for moisture. These chemical groups in the fibers readily bond with water molecules through hydrogen bonding, which causes the fibers to swell and absorb more moisture. However, composites treated with alkali show significantly less moisture uptake. This improvement mainly comes from the removal of hemicellulose and partial removal of lignin from the fiber surface, which reduces the number of available hydroxyl groups that attract water. Alkali treatment also increases the cellulose crystallinity within the fibers, creating a tighter, more compact structure that resists water penetration. Additionally, the treatment enhances the bond between the fibers and the polymer matrix, reducing gaps where water could otherwise seep in. The work by Sugiman et al. [48] demonstrates that water uptake significantly decreases with increasing sodium hydroxide (NaOH) concentrations used in the alkali treatment. Specifically, under room temperature (28 °C), a 12% NaOH treatment reduces water absorption by approximately 51% compared to untreated composites. The improvement is attributed to removing hemicellulose and lignin, which are more hydrophilic and contribute to water sorption.

#### 3.1.2. Adding Filler Materials

**Figure 1:**
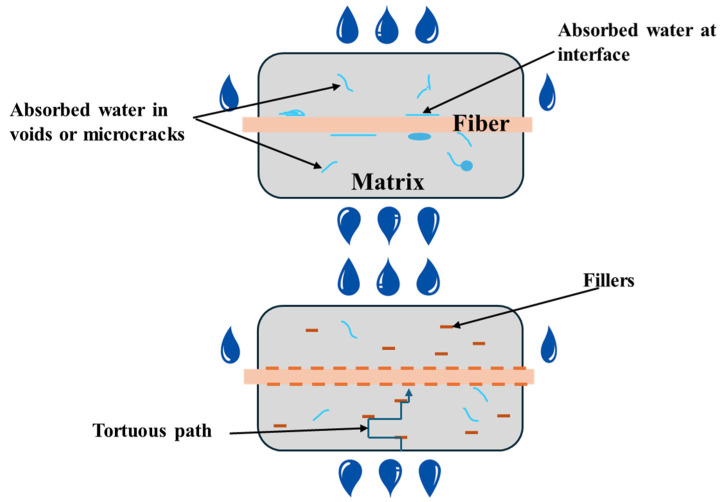
Schematic illustration of the physical effects of moisture on unfilled and filled composites.

Adding filler materials to bamboo fiber-reinforced polymer composites can significantly reduce water uptake through several mechanisms. Bamboo fibers are inherently hydrophilic, meaning they readily absorb moisture, which can lead to swelling, degradation, and reduced mechanical performance of the composite. As shown in Figure 1, when fillers are incorporated into the matrix, they help to occupy the microvoids and gaps that typically exist at the fiber–matrix interface. This reduces the number of pathways available for water molecules to infiltrate the composite structure. Additionally, fillers enhance the overall packing density of the material, thereby decreasing the free volume and making it more difficult for water to diffuse through the matrix. Many fillers, especially those with a platelet or flake-like morphology, act as physical barriers by creating a tortuous path that impedes water migration [60]. Moreover, if the fillers are hydrophobic or surface-treated to repel water, they can lower the number of hydrophilic sites within the composite, further minimizing moisture absorption [61]. The presence of fillers can also improve the interfacial adhesion between the bamboo fibers and the polymer matrix, leading to fewer weak spots where water might penetrate [62].

Recent work by Chakkour et al. [30] investigated the influence of montmorillonite (MMT) and eggshell powder (ESP) fillers on the water absorption behavior of bamboo fiber-reinforced epoxy composites. The study finds that unfilled composites absorb a high amount of water, up to 16.46% after immersion, while adding fillers like MMT and ESP significantly lowers this absorption. Among them, composites with 3 wt% MMT show the most significant improvement, reducing water uptake by 37.8%, and those with 3 wt% ESP achieve a 25.7% reduction. These benefits come from the fillers acting as barriers, filling gaps between fibers and the matrix, and improving the bond between them. In the study by Tahir et al. [50], the water absorption of bamboo fiber/epoxy composites is notably reduced by adding silicon carbide (SiC) fillers. When 2 to 6 wt% SiC is incorporated, water uptake drops by 58% to 67%. The hybrid composite with 6 wt% SiC has the lowest water absorption, thanks to SiC’s hydrophobic nature and its ability to fill voids within the matrix. This densification limits water’s pathways to enter the material, significantly improving its moisture resistance. In the work by Gupta [63], ceramic fillers, viz., aluminum oxide and silicon carbide, are added to short bamboo fiber-reinforced epoxy composites to explore the effects of ceramic fillers on water absorption. Adding aluminum oxide and silicon carbide fillers significantly reduces the water absorption. The water absorption rate for an unfilled composite after 60 days is 9.2%, whereas the inclusion of aluminum oxide and silicon carbide fillers reduces it to 6.8%. Filler particles diminish the void fraction of composites, which reduces their water absorption ability.

#### 3.1.3. Hybridization

Hybridization decreases the water uptake of bamboo fiber-reinforced polymer composites [64]. When bamboo is hybridized with less hydrophilic or hydrophobic fibers, viz., glass, carbon, or synthetic fibers, the overall number of water-attracting sites in the composite is reduced. This combination lowers the composite’s tendency to absorb water. Moreover, hybridization often enhances the fiber–matrix interfacial bonding, especially if the added fibers are more compatible with the matrix or are surface-treated [65]. A stronger interface minimizes the presence of microvoids and gaps, which otherwise serve as pathways for water ingress. Additionally, hybrid fibers can act as a physical barrier that slows down or restricts the diffusion of water into the composite [66]. This barrier effect is particularly notable in composites incorporating impermeable fibers like glass or carbon. Hybridization also contributes to a more optimized and densely packed composite structure, reducing porosity and further limiting moisture penetration [67].

The study by Mim [68] investigates the water absorption behavior of bamboo–glass fiber-reinforced hybrid composites immersed in a 3.5% NaCl solution for 21 days, simulating marine conditions. Quantitatively, the bamboo-reinforced epoxy composite exhibits a water absorption of 7.36%. In contrast, bamboo–glass fiber reinforced hybrid composites demonstrate the lowest absorption at 5.05%, benefiting from glass fiber reinforcement. In the study conducted by Alfeki and Feyissa [69], significant emphasis is placed on evaluating the water absorption characteristics of bamboo fiber-reinforced polyester composites modified with chopped glass fiber. The results reveal that incorporating chopped glass fiber reduces water absorption in composites. For 40 wt.% bamboo fiber composites, the recorded water absorption value is 2.6%. Adding 5 and 10 wt.% chopped glass fiber, the water absorption drops significantly to 1.78 and 1.65%, respectively. The study by Ahmad et al. [51] explores the water absorption behavior of bamboo–glass fiber-reinforced hybrid epoxy composites. Bamboo fiber–epoxy composite shows the highest water uptake, reaching approximately 1.75% at the end of the testing period. In comparison, the bamboo–glass fiber hybrid composite shows a water uptake of 1.57%. Hybridizing bamboo fibers with glass fibers reduces the overall hydrophilicity of the composite, resulting in less water absorption.

## 4. Effect of Water-Soaking Conditions on Mechanical Properties of Bamboo Fiber-Reinforced Polymer Composites

**Figure 2:**
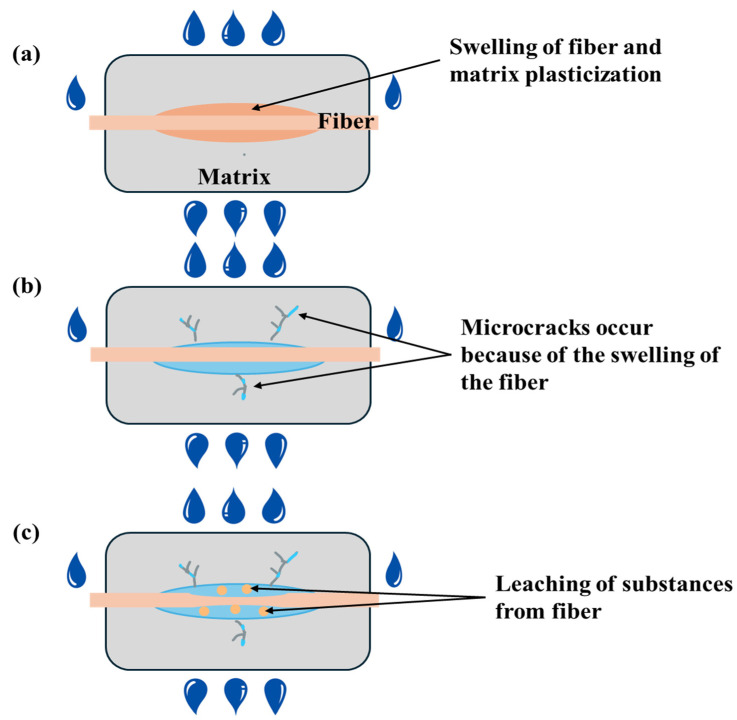
Water-soaking aging mechanisms of bamboo fiber polymer composites (**a**) Swelling of fiber, (**b**) Microcracking of matrix, (**c**) Leaching of substances.

The water-soaking effect on bamboo fiber-reinforced polymer composites is a significant concern, especially for applications exposed to humid or wet environments. Figure 2 presents the water-soaking aging behavior of bamboo fiber composites. During water-soaking, water molecules infiltrate the polymer matrix and diffuse through capillary action along the fiber–matrix interface, accompanied by moisture absorption into the fibers themselves [70]. This results in the swelling of fibers and matrix plasticization, as depicted in Figure 2a. When swelling becomes significant, it can cause the matrix to crack around the fibers, leading to permanent mechanical degradation.

Furthermore, as shown in Figure 2b, the fibers may split due to differential swelling stresses between the inner cell wall layers. Simultaneously, sections within the bamboo fibers that are poorly crystallized, such as amorphous cellulose, hemicellulose, pectins, and waxes, begin to leach out over time. This leaching, illustrated in Figure 2c, contributes to fiber–matrix debonding and irreversible structural changes. This compromised bond reduces stress transfer efficiency from the matrix to the reinforcing fibers, which is crucial for maintaining strength and stiffness in composite materials. Consequently, even under moderate loading, the water-aged composites show reduced tensile, flexural, and impact properties compared to their unaged counterparts. Table 3 presents the percentage of reduction in mechanical properties of water-soaked specimens compared to unaged specimens.

**Figure 3:**
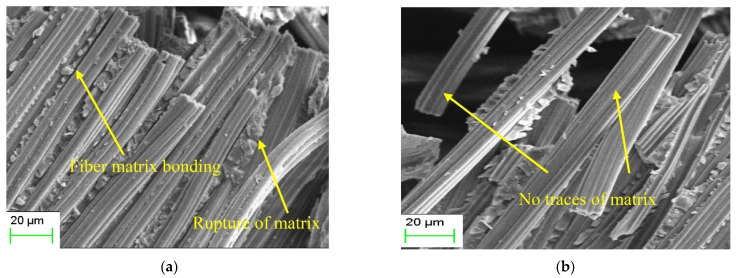
SEM images of (**a**) unaged composites and (**b**)water-soaked specimens [51].

The SEM images (Figure 3) illustrate the fracture surfaces of bamboo fiber-reinforced polymer composites under unaged and water-soaked conditions. In the unaged sample (Figure 3a), the fibers exhibit clear signs of strong fiber–matrix adhesion, with remnants of the polymer matrix still attached to the fiber surfaces. This suggests adequate interfacial bonding, which is crucial for efficient stress transfer between the matrix and the reinforcement. The presence of matrix rupture rather than clean fiber detachment indicates that failure occurred cohesively within the matrix, demonstrating the structural integrity of the interface. In contrast, the water-soaked specimen (Figure 3b) shows a significant degradation in interfacial bonding. The fibers appear clean, with no traces of matrix adhesion, suggesting that prolonged water exposure has led to fiber–matrix debonding. This deterioration is likely due to moisture-induced effects such as swelling, hydrolysis, or polymer plasticization, which reduce adhesion strength. The smooth, aligned fibers imply a fiber pull-out failure mechanism, typically associated with weak interfacial bonding. Overall, the comparison highlights the detrimental effect of water absorption on the interfacial integrity and mechanical reliability of natural fiber composites.

### 4.1. Mitigation Strategies for Reducing the Impact of Water-Soaking Conditions on the Mechanical Properties of Bamboo Fiber-Reinforced Polymer Composites

#### 4.1.1. Surface Treatment of Bamboo Fibers

The interfacial bonding between bamboo fibers and the polymer matrix is governed by mechanical interlocking and chemical interactions, which are critical for effective load transfer and long-term durability of the composite. Bamboo fibers are composed mainly of cellulose (50–60%), hemicellulose (20–25%), and lignin (15–20%), all of which are rich in hydroxyl (−OH) groups that make the fiber surface highly polar and hydrophilic. This natural polarity creates poor compatibility with hydrophobic polymer matrices such as epoxy, polypropylene, or polyethylene. To address this issue, various surface treatments, including alkali treatment, silane coupling, and acetylation, are employed to modify the fiber surface. These treatments remove amorphous hemicellulose, partially remove lignin, and expose more crystalline cellulose, thereby reducing hydrophilicity and enhancing surface roughness for better mechanical interlocking. Among them, silane coupling agents are especially effective as they form covalent bonds with the hydroxyl groups on the fiber and also react with functional groups in the polymer matrix, acting as molecular bridges that significantly improve interfacial adhesion. In the case of epoxy matrices, chemical interactions may also involve hydrogen bonding and covalent bonding between matrix epoxide groups and treated fiber surfaces. These improved interactions help reduce fiber pull-out, enhance interfacial shear strength, and increase resistance to environmental degradation, ultimately improving the mechanical performance and durability of bamboo fiber-reinforced polymer composites.

Prabhu et al. [71] explored the influence of the alkaline treatment of bamboo fiber on the effect of water absorption on the mechanical properties of bamboo fiber-reinforced epoxy composites. Composite reinforced with 5% NaOH-treated fiber shows better performance when compared with untreated fiber-reinforced composites. Alkaline treatment of the fiber has reduced the impact of water absorption on mechanical properties. The tensile and flexural strengths of treated fiber composites decline by 10% and 9.8% under water-soaking conditions, whereas untreated fiber composites show a decline of 18.12% and 20.08%. In the work of Chakkour et al. [30], long-term water-aging effects on the durability of alkali-treated bamboo fiber-reinforced composite are evaluated. The fibers are treated in 1 wt.% sodium hydroxide (NaOH) solution for 0.5 h at 25 °C. Untreated fiber composites lose about 18.15 and 18.55%, while treated fiber specimens lose 15.79 and 14.47% of their unaged tensile strength and Young’s modulus after attaining 120 days of aging.

The study by Zhang et al. [52] presents an extensive assessment of the influence of water absorption on the mechanical performance of treated and untreated bamboo fiber-reinforced polybenzoxazine composites. The fibers are treated with 6 wt.% sodium hydroxide (NaOH) solution. Tensile strength in treated fiber composites reduces by 65% when exposed to water-soaking at 80 °C for 20 days, whereas composites without treatment have a greater reduction of 72.5%. This increase in treated composites is primarily attributed to increased bonding among the fibers and the polymer matrix.

In a study by Chunhong et al. [76], the influence of alkali–silane treatment and hygrothermal aging on bamboo fiber polypropylene composites is investigated. The treatment consists of initially immersing the fibers in a 5% alkali solution, followed by NaOH treatment for 1 h at room temperature. The composites are subsequently subjected to hygrothermal aging in a controlled environment maintained at 40 °C and 93% humidity for 60 days. Results indicate that in these conditions, treated composites’ flexural and shear strengths decrease by 14% and 12%, respectively, while untreated composites experience greater decreases of 19.23% and 14.28%.

#### 4.1.2. Matrix Modification by Adding Filler Materials

Matrix modification by incorporating filler materials is a viable approach to alleviate the negative consequences of water aging on the mechanical properties of bamboo fiber-reinforced polymer composites. Water aging generally causes moisture uptake, contributing to swelling, debonding between fibers and matrix, and substantial mechanical strength and stiffness loss. However, adding fillers in the polymer matrix solves these problems by modifying the structural and barrier characteristics of the composite. Fillers enhance the interfacial adhesion between the bamboo fibers and the polymer matrix. Increasing adhesion at this key interface reduces the fiber pull-out and debonding tendency upon exposure to water. This enhanced adhesion maintains the mechanical stability of the composite despite long exposure times to moisture.

The work by Sugiman et al. [48] explores the influence of nano CaCO_3_ on the mechanical properties of unaged and aged bamboo fiber unsaturated polyester composites. The unsaturated polyester matrix is modified by adding nano CaCO_3_ with varying wt.%, i.e., 1, 3, and 5. The specimens are aged in distilled water for 112 days. Compared to the unaged composites, bamboo fiber composites’ tensile and flexural strengths are decreased by 47% and 58%, respectively. The addition of 1, 3, and 5 wt.% nano CaCO_3_ decline the reduction in the tensile strength of bamboo fiber–nano CaCO_3_-modified unsaturated polyester composites to 43, 37, and 37%, respectively. Similarly, the flexural strength of bamboo fiber–nano CaCO_3_-modified unsaturated polyester composites declines to 52, 53, and 42% for the nano CaCO_3_ contents of 0, 1, 3, and 5 wt.%, respectively. This improvement of aged composites is likely due to the reduced water absorption after nano CaCO_3_ addition, which reduces the degradation of the bamboo fiber unsaturated polyester matrix interfaces.

The recent work by Ahmad et al. [51] investigates the influence of nanoclay on the tensile and flexural strengths of unaged and aged bamboo fiber–epoxy composites. The epoxy resin is modified by adding 2 wt.% of nanoclay. The composites are aged in tap water for 84 days. Compared to unaged composites, the tensile and flexural strengths of aged bamboo fiber–epoxy composites decrease by 13.86% and 12.9%, respectively. Meanwhile, the addition of nanoclay decreases the percentage of reduction in tensile and flexural strengths of aged composites to 11% and 10%, respectively. Another work by Ahmad et al. [75] explores the influence of nanoclay on the tensile and flexural strengths of unaged and aged bamboo fiber–epoxy composites. The epoxy resin is modified by adding 2 wt.% of nanoclay. The composites are aged in boiling water, which means that samples are placed in boiling water for two hours and then kept outside for 22 h. These steps are repeated daily for ten days, resulting in a total boiling time of twenty hours. Compared to unaged composites, the tensile and flexural strengths of aged bamboo fiber–epoxy composites decrease by 27% and 20%, respectively. Meanwhile, the addition of nanoclay decreases the percentage of reduction in tensile and flexural strengths of aged composites by 21% and 14%, respectively. Adding nanoclay can reduce the negative effects of water soaking on tensile strength by acting as a barrier to water diffusion, enhancing interfacial adhesion, imparting hydrophobicity, and lowering matrix swelling.

#### 4.1.3. Hybridization of Fibers

The hybridization of fibers has been shown to significantly reduce the impact of aging on the mechanical properties of bamboo fiber-reinforced polymer composites. Incorporating other fibers into the composite greatly improves the overall resistance to moisture ingress and environmental degradation. These secondary fibers, typically more hydrophobic and dimensionally stable, help limit water absorption and serve as barriers that reduce moisture diffusion to the bamboo fibers. Additionally, hybridization facilitates better load distribution among the reinforcing phases, reducing stress concentrations on the bamboo fibers and thereby mitigating crack initiation and propagation caused by environmental factors.

In the work, Mim [68] studies the influence of the hybridization of glass fiber on the tensile and flexural strengths of unaged and aged bamboo fiber–epoxy composites. The pure bamboo fiber composite (B–B–B–B–B) demonstrates moderate mechanical strength, achieving 40.7 MPa in tensile strength and 77.2 MPa in flexural strength. When bamboo is hybridized with glass fibers, especially in the G-B-B-B-G configuration (glass–bamboo–bamboo–bamboo–glass), there is a dramatic improvement, with tensile strength reaching 87.1 MPa and flexural strength 170.7 MPa. This reflects a 110% increase in tensile strength and 121% in flexural strength compared to the bamboo-only composite. These enhancements are attributed to glass fibers’ high stiffness and load-bearing capacity and bamboo fibers’ moderately high mechanical contribution, especially when arranged in a bidirectional layout. The specimens are immersed in NaCl water at 25 °C for 21 days. Compared to unaged composites, the tensile and flexural strengths of aged bamboo fiber–epoxy composites decrease by 15.48% and 40.8%, respectively. Meanwhile, hybrid glass–bamboo fiber–epoxy composites decrease the percentage of reduction in tensile and flexural strengths of aged composites by 12.06% and 35.51%, respectively.

The study by Haseebuddin et al. [73] explores the influence of hybridizing bamboo mat with varying proportions of chopped waste glass fiber mats in a polyester resin matrix, which is thoroughly investigated, particularly focusing on flexural strength and resistance to water-induced aging. The base composite consisting of 50 wt.% bamboo mat and 50 wt.% polyester resin (without any glass fiber) serves as the reference. To enhance performance, glass fiber mats are incorporated in increasing proportions, 10, 20, and 30 wt.%, while proportionally reducing both the bamboo and resin content to maintain a consistent total weight. The flexural strength of the composites improves significantly with increasing glass fiber content. The bamboo–polyester composite without glass fiber exhibits a flexural strength of approximately 140 N/mm^2^. Upon introducing 10 wt.% glass fiber, the flexural strength rises to around 200 N/mm^2^, showing a marked increase due to the added stiffness and resistance to shear that the glass fibers provide. The strength further increases to 270 N/mm^2^ with 20 wt.% glass fiber and peaked at about 360 N/mm^2^ with 30 wt.% glass fiber. This improvement is attributed to the high modulus of glass fiber, better stress distribution, enhanced matrix–fiber bonding, and the increased rigidity imparted by the synthetic reinforcement. The sandwich structure with bamboo and glass fiber layers helped arrest crack propagation and improved interfacial adhesion, thereby elevating the load-bearing capacity. The resistance of the hybrid composites to hygrothermal aging is also evaluated through immersion tests in water for 15 and 30 days, simulating moisture exposure. Results show that the bamboo–polyester composite without glass fiber suffered a 33% reduction in flexural strength after 30 days, dropping from ~140 N/mm^2^ to ~94 N/mm^2^. This severe degradation is due to the hydrophilic nature of bamboo fibers, whose lignocellulosic structure attracts moisture, leading to fiber swelling, matrix debonding, and overall property deterioration. In contrast, the composite containing 30 wt.% glass fiber shows only an 11% decrease, with flexural strength reducing from ~360 N/mm^2^ to ~320 N/mm^2^ after 30 days of water exposure. Similarly, composites with 10% and 20% glass fiber show intermediate reductions in strength, around 24% and 17%, respectively.

Ahmad et al. [51] studied the influence of bamboo and glass fiber hybridization on the tensile and flexural strengths of unaged and aged epoxy-based composites. The composites are aged in tap water for 84 days. Compared to unaged composites, the tensile and flexural strengths of aged bamboo fiber–epoxy composites decreased by 13.86% and 12.9%, respectively, whereas the tensile and flexural strengths of the aged bamboo–glass fiber hybrid composite samples show a reduction of 10% and 9.3% when compared to the unaged composite. Glass fibers can enhance a composite’s mechanical properties, such as its resistance to deformation and cracking. The improved interfacial bonding between the fiber and matrix can enhance the overall integrity of the composite material. These enhancements can reduce the propensity for fiber–matrix debonding and damage, which can occur due to water absorption; thus, the reduction in tensile strength is restricted.

## 5. Effect of Different Types of Aging Conditions on Mechanical Properties of Bamboo Fiber-Reinforced Polymer Composites

Table 4 provides a comparative overview of the degradation in mechanical properties of bamboo fiber-reinforced polymer composites under various aging conditions.

The work by Chunhong et al. [76] explores the effect of hygrothermal chamber aging on untreated and treated bamboo fiber–polypropylene composites. The hygrothermal aging composites’ properties are tested in an environmental testing chamber at 40 °C and 93% RH, respectively, for 60 days. At the end of hygrothermal aging, the untreated fiber composites display tensile, flexural, and shear strengths of 32, 41, and 4.5 MPa, respectively. Meanwhile, the alkali–silane-treated fiber composites exhibit improved tensile, flexural, and shear strengths of 37, 60, and 6.4 MPa, respectively. After the alkali–silane treatment, the polarity of the bamboo fibers decreases, leading to reduced surface-active groups and a difference in expansion between the fibers and the matrix.

In the study by Fei et al. [77], the flexural performance of bamboo fiber-reinforced high-density polyethylene composites is evaluated under artificial weathering conditions, with and without the incorporation of nano-TiO_2_ particles. Two composites are studied: the unmodified bamboo composite (BH) and one modified with nano-TiO_2_ (BHTi). The two samples are subjected to xenon-arc accelerated weathering for four weeks to simulate long-term environmental effects. The findings indicate that flexural strength decreases in both composites with time, primarily due to photo-oxidative deterioration leading to fiber–matrix interface degradation, microcrack development, and general structure weakening. After four weeks, the flexural strength of the BH composite reduces by 24%, whereas the BHTi composite reduces slightly by 15%. This enhanced resistance in the nano-TiO_2_-treated composite is due to the nanoparticles’ capacity to absorb and scatter UV light, safeguarding the polymer chains against degradation and protecting the bamboo fibers. Due to this, the aging and degradation mechanism is reduced, increasing the composite’s durability.

Fajardo Cabrera de Lima et al. [78] assessed the mechanical properties of polypropylene/bamboo fiber (PP/BF) composites under natural outdoor weather exposure for 12 months. The research centers around tensile and impact strengths, specifically looking at how coupling agents (CAs) affect durability. Two coupling agents are tested: maleic anhydride (MA), a petrochemical-derived agent, and citric acid (CI), a naturally sourced alternative. All composites undergo a steady reduction in tensile and impact strengths throughout the exposure time. The pure composite without the coupling agent (PP/BF) has the most significant decline, with tensile strength being reduced by 37% and impact strength by 29%. Composites with coupling agents retained more mechanical properties: tensile and impact strengths of the PP/BF/MA composite were reduced by 17% and 25.3%, respectively. By contrast, the PP/BF/CI composite demonstrates reductions of 21.4% and 24.7%. These result from enhanced fiber–matrix adhesion from the coupling agents, allowing for better energy dissipation, crack initiation, and growth delay when subjected to mechanical stress.

The study by Jiang et al. [79] investigates the effects of steam aging and boron nitride filler on the mechanical properties of bamboo fiber-reinforced high-density polyethylene composites. The composites are made without and with boron nitride filler and steam-aged for periods of up to 20 days at 99 °C. Tensile and flexural strength is found to degrade notably after 20 days of steam aging due to fiber swelling, hydrolysis, and interface degradation. The tensile and flexural strength of bamboo fiber-reinforced high-density polyethylene composites decrease by 39.7 and 39.6%, respectively, upon steam aging. The incorporation of 6 wt.% boron nitride reduces the steam-aging effect. Both tensile and flexural strengths of composites filled with boron nitride decrease by 19.5 and 18.5%, respectively. The improvement is attributed to adding boron nitride, which can slow the damage process of steam aging on the fiber/matrix interface.

The work by Peng et al. [80] explores the UV aging resistance properties of bamboo fiber/HDPE composites through silane coupling agent-modified nano-SiO_2_-TiO_2_. Accelerated UV aging tests are performed for up to 1200 h using a UV-A lamp, simulating long-term exposure. The unmodified specimen exhibits a marked degradation in flexural strength, dropping from 36.55 MPa to 30.88 MPa, representing a 15.6% reduction. In contrast, the modified composites show a significantly lower decline. For instance, the composite with 1 wt.% KH550/SiO_2_-TiO_2_ display only a 10.1% decrease (from 39.76 MPa to 35.73 MPa), while the 2 wt.% sample drop by 9.43%. Most notably, the composite containing 3 wt.% KH550/SiO_2_-TiO_2_ experiences a 6.36% reduction in flexural strength, from 43.39 MPa to 40.63 MPa, after 1200 h of UV aging. This result underscores the improved UV shielding and photostability of the hybrid nanofiller. The enhancement is attributed to the UV-absorbing properties of TiO_2_, the reduction in photocatalytic degradation by the SiO_2_ coating, and the improved nanoparticle dispersion and bonding facilitated by the silane coupling agent KH550.

The investigation by Rao et al. [72] explores the degradation behavior of bamboo fiber-reinforced polylactic acid composites under different environmental aging conditions, including soil burial and refrigerator storage. During soil burial, the mechanical properties of composites exhibit a consistent decline over the aging period due to environmental biodegradation. After 28 days, the tensile strength drops from 34 MPa to 11 MPa, showing a 67.64% reduction. Simultaneously, the flexural strength decreases from 87.2 MPa to 43.70 MPa, reflecting a 49.88% decline. This deterioration is attributed to the degradation of the matrix in the moist soil environment and the swelling of bamboo fibers, which weakened the interfacial bonding between fiber and matrix. In contrast, refrigerator aging has a comparatively milder impact. After four weeks at temperatures below 4 °C, the tensile strength decreases to 18 MPa, indicating a 47.05% reduction, while the flexural strength declines to 65.50 MPa, equating to a 21.70% drop. The low-temperature environment slows the degradation rate and limits moisture uptake, resulting in less fiber swelling and interfacial debonding than soil burial.

The work by Li and Li [81] investigates the effect of freeze–thaw aging conditions on the mechanical properties of bamboo fiber/polypropylene composites. The composites are subjected to accelerated freeze–thaw aging consisting of ten cycles, each involving 24 h of water immersion at 23 ± 2 °C, followed by 24 h of freezing at −27 ± 2 °C, and a final 24 h of thawing at room temperature. The total exposure time amounts to 720 h. By the end of the 720 h exposure period, the tensile, flexural, and impact strength retention rates are 76.90, 86.89, and 71.83%, respectively. The mechanisms behind these declines in mechanical performance are closely associated with moisture absorption and differential thermal expansion. Initially, the polypropylene matrix effectively encapsulated the bamboo fibers, preventing moisture ingress. However, as the aging process progresses, the degradation of the polymer matrix allows water to penetrate and accumulate at the fiber–matrix interface. During freezing, this water expands, creating internal stresses and microcracks. Upon thawing, capillary effects draw additional moisture into these cracks, promoting further damage. This cyclic stress eventually leads to the formation of internal voids, delamination, and severe loss of mechanical integrity.

## 6. Molecular-Level Degradation Mechanisms in Bamboo Fiber-Reinforced Polymer Composites

Bamboo fiber-reinforced polymer composites undergo several chemical degradation processes when exposed to environmental aging conditions such as moisture, UV radiation, and elevated temperatures. These molecular-level changes compromise the structural integrity, interfacial bonding, and overall durability of the composites. The key degradation mechanisms are outlined below:

Cellulose hydrolysis: Under prolonged exposure to water or humid environments, hydroxyl groups in cellulose and hemicellulose interact with water molecules through hydrogen bonding. This leads to the hydrolytic cleavage of glycosidic linkages, particularly in the amorphous regions of cellulose. The breakdown of these polysaccharide chains causes fiber swelling, microstructural loosening, and reduced mechanical strength.

Oxidative degradation of the polymer matrix: Thermal and UV aging generate free radicals within the polymer matrix (e.g., epoxy or polyester). These reactive species initiate chain scission, oxidation of functional groups, and cross-linking density reduction. Over time, the matrix becomes brittle, loses ductility, and exhibits microcracking and surface embrittlement.

Photochemical destruction: Exposure to UV radiation leads to photolytic cleavage of chemical bonds in the bamboo fiber’s polymer matrix and lignin. This degradation pathway causes discoloration, surface erosion, and the breakdown of aromatic structures, especially in lignin, which is highly sensitive to UV light.

Leaching of hemicellulose and extractives: During prolonged water immersion or soil burial, low-molecular-weight components such as hemicellulose, pectins, and waxes leach out from the fiber surface. This leaching weakens interfacial adhesion between the fiber and matrix, increases void content, and accelerates further moisture diffusion.

Synergistic effects: In many real-world applications, composites are simultaneously exposed to multiple stressors (e.g., UV + moisture). These combined conditions intensify degradation rates through synergistic interactions, such as accelerated oxidation in moist-UV environments.

**Figure 4:**
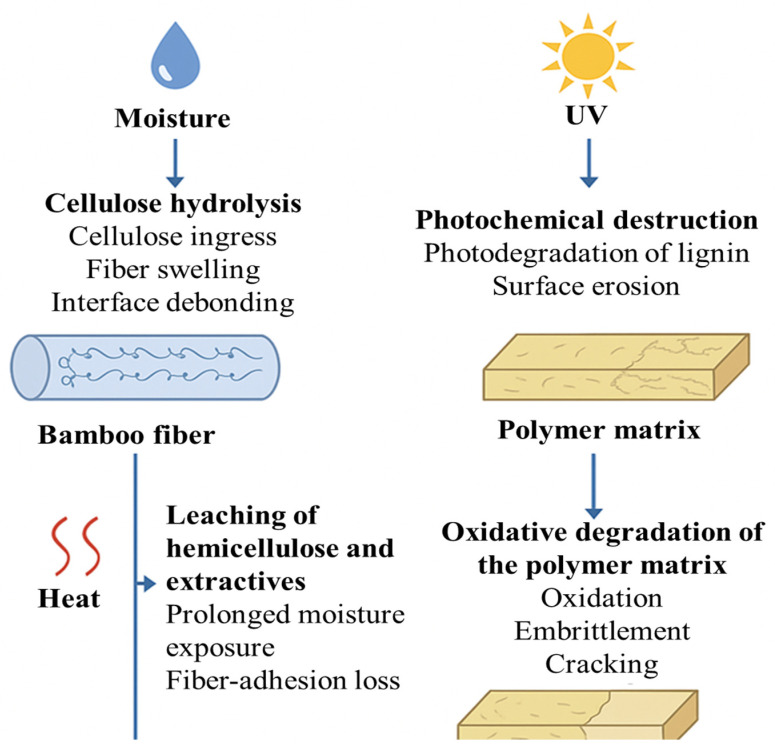
Schematic representation of molecular degradation in bamboo fiber-reinforced composites under aging conditions.

Figure 4 schematically illustrates these degradation pathways, showing how moisture, heat, and UV radiation induce chemical breakdown in the composite’s fiber and matrix phases.

## 7. Application of Bamboo Fiber-Reinforced Polymer Composites

Table 5 correlates application areas of bamboo fiber composites with relevant aging conditions, their effects, and corresponding mitigation strategies.

While Table 5 summarizes application areas along with typical aging conditions and mitigation strategies, the following section extends those insights by recommending appropriate composite compositions for each condition:

- Outdoor construction panels and cladding: These applications face frequent exposure to moisture, UV radiation, and temperature variations. Bamboo fibers treated with NaOH or silane, combined with nanoclay- or TiO_2_-filled epoxy or HDPE matrices, offer improved resistance to environmental aging. Hybridization with glass fibers further enhances structural integrity and reduces water uptake, as shown in studies by Ahmad et al. [51] and Peng et al. [80].
- Automotive interiors: Interiors are exposed to humidity and thermal cycling. For such conditions, epoxy or polypropylene matrices reinforced with alkali-treated bamboo fibers and nanoclay or CaCO_3_ fillers demonstrate stable mechanical properties over time. Sugiman et al. [48] and Ahmad et al. [51] have shown that these formulations limit fiber swelling and matrix degradation under prolonged exposure.
- Marine and high-humidity environments: Composites incorporating bamboo–glass fiber hybrids within epoxy matrices are more suitable, as glass fibers reduce overall hydrophilicity and enhance dimensional stability. Mim [68] reports that such hybrids show significantly lower water absorption and degradation in saline immersion tests.
- Eco-friendly packaging and consumer goods: Composites made from enzyme- or untreated bamboo fibers and biodegradable polymer matrices such as PLA or PHBV are recommended for applications emphasizing end-of-life biodegradability. While these systems may offer reduced mechanical performance, they perform well in soil burial or refrigerated storage, as highlighted by Rao et al. [72].
- Building insulation panels: These face hygrothermal aging. Composites fabricated with alkali-treated bamboo fibers and hydrophobic fillers like silicon carbide or nanoclay help retain mechanical and thermal insulation properties. Work by Chakkour et al. [30] and Ahmad et al. [51] supports the effectiveness of such modifications.

## 8. Conclusions

Bamboo fiber-reinforced polymer composites offer a sustainable alternative to synthetic composites due to their biodegradability, affordability, and acceptable mechanical performance. However, their susceptibility to environmental aging, such as water immersion, hygrothermal exposure, UV radiation, soil burial, and freeze–thaw cycles, significantly limits their long-term durability and applicability.

Among all aging factors, moisture absorption is the most critical degradation mechanism. The hydrophilic nature of bamboo fibers, due to abundant hydroxyl groups, leads to water uptake, fiber swelling, interfacial debonding, and a cascade of mechanical property losses, including tensile, flexural, and impact strength.

The review highlights that various mitigation strategies can significantly enhance the durability of these composites:

- Surface treatments such as alkali and silane modification effectively reduce fiber hydrophilicity, improve fiber–matrix adhesion, and help retain mechanical performance under aging.
- Filler additions enhance the matrix’s barrier properties by reducing voids and increasing tortuosity for water diffusion. These improvements contribute to reduced moisture ingress and better retention of strength.
- Hybridization with synthetic fibers like glass reduces overall water absorption, improves dimensional stability, and provides better stress distribution. Hybrid composites exhibit notably higher durability and reduced property degradation under environmental stresses.

The severity of mechanical degradation varies with the aging condition. Soil burial and prolonged water immersion at elevated temperatures cause the most pronounced damage, while refrigeration has the least effect due to minimal moisture activity. UV exposure and hygrothermal aging also lead to considerable degradation in the absence of protective modifications.

In summary, although bamboo fiber composites inherently face durability challenges, appropriate treatments and material modifications can significantly extend their service life. With these enhancements, they show strong potential for sustainable use in automotive, construction, furniture, and packaging applications where environmental resilience is critical.

## Figures and Tables

**Table 1:** Different methods used to fabricate bamboo fiber-reinforced polymer composites.

Method	Fiber Orientation	Polymer Type	Ref.
Hand lay-up	Chopped	Unsaturated polyester resin	[26]
Compression molding	Randomly oriented fibers	Polyester resin	[27]
Hand lay-up and compression molding	Unidirectional fibers	Epoxy resin	[28,29,30]
	Bidirectional fibers	Epoxy resin	[31]
Injection molding	Short fibers	Polypropylene pellets	[32]
	Chopped fibers	HDPE	[33]
	Chopped fibers	PE, PP, PA12, ABS, PA6, and Durabio	[34]
General casting	Fiber powder	Epoxy resin	[35]
Hot pressing	Cross-ply (0°/90°) orientations	MHU resin	[36]
	Chopped fibers	Epoxy resin	[37]
Dry blend rotational molding	Fiber powder	Linear low-density polyethylene powder	[38]
Vacuum bag molding	Bidirectional fiber mat	Vinyl ester resin	[39,40]
Vacuum-assisted resin transfer molding	Randomly oriented fibers	Epoxy resin	[41]
Extrusion method	Fiber powder	Polyester resin	[42]

**Table 2:** Water uptake (%) by different bamboo fiber-reinforced polymer composites.

Type of Water Soaking	Temperature	Duration	Bamboo Fiber (wt.%)	Polymer Type	Water Uptake (%)	Ref.
Tap water	Ambient	10 Days	20 wt.%	Epoxy resin	2.3%	[50]
Tap water	Ambient	84 Days	50 wt.%	Epoxy resin	2.2%	[51]
Tap water	25 °C	20 Days	20 wt.%	Benzoxazine resin	4.24%	[52]
	80 °C				6.32%	
Water	Ambient	20 Days	75 wt.%	Epoxy resin	3.5%	[45]
	50 °C				6.6%	
	80 °C				7.8%	
Water	Ambient	5 Days	40 vf.%	Epoxy resin	11%	[53]
Distilled water	Ambient	11 Days	20 wt.%	Polystyrene resin	13.59%	[54]
Distilled water	Ambient	10 Days	20 wt.%	Poly(hydroxybutyrate-co-valerate)	2.25%	[55]

**Table 3:** Percentage of reduction in mechanical properties of water-soaked specimens compared to unaged specimens.

Type of Water Soaking	Bamboo FIBER (wt.%)	Polymer Type	% of Reduction in Mechanical Properties				Ref.
			Tensile Strength	Flexural Strength	Impact Strength	Hardness	
Tap water at room temperature	50 wt.%	Epoxy resin	13.86%	12.9%	-	-	[51]
Tap water at 25 °C and 80 °C	20 wt.%	Benzoxazine resin	59% and 72.5%	25.72% and 26.02%	-	-	[52]
Distilled water at room temperature	50 wt.%	Epoxy resin	18.12%	20.08%	10.11%	21.42%	[71]
Distilled water at room temperature	30 wt.%	Epoxy resin	18.15%	-	-	-	[30]
Water	20 wt.%	PLA	73.5%	45.15%	-	-	[72]
Water	50 wt.%	Polyester resin	-	33%	-	-	[73]
Distilled water	25 vf.%	Polyester resin	47%	58%	-	-	[48]
NaCl water at 25 °C	5 layers	Epoxy resin	15.48%	40.8%	-	-	[68]
Water at 100 °C	42 wt.%	Epoxy resin	57%	62%	-	-	[74]
Boiling water	50 wt.%	Epoxy resin	27%	20%	-	-	[75]

**Table 4:** Percentage of reduction in mechanical properties of different aged specimens compared to unaged specimens.

Types of Aging Conditions	Parameters	Bamboo Fiber (wt.%)	Polymer Type	% of Reduction in Mechanical Properties				Ref.
				Tensile Strength	Flexural Strength	Impact Strength	Hardness	
Hygrothermal chamber aging	40 °C and 93% RH	60 wt.%	Polypropylene	18.42%	19.23%	-	-	[76]
Accelerated weathering	Xenon-arc radiation at 65 °C and 18 min water spray.	36 wt.%	High-density polyethylene	-	24%	-	-	[77]
Natural outdoor aging	Temp: 11 to 36 °C Average rainfall: 6.6 mm UV Index: 4 to 14	30 wt.%	Polypropylene	37%	-	29%	-	[78]
Steam aging	Steam is generated by heated distilled water,	44 wt.%	High-density polyethylene	39.7%	39.6%	-	-	[79]
Ultraviolet aging	UV energy density: 0.76 W/m^2^, Temp: 0 °C ± 3 °C	50 wt.%	High-density polyethylene	-	15.6%	-	-	[80]
Soil burial	Garden soil with some moisture content, 28 days	20 wt.%	Polylactic acid	67.64%	49.88%	-	-	[72]
Refrigerator aging	Temp: below 4 °C, 28 days			47.05%	21.70%	-	-	
Freeze–thaw cycle aging	10 freeze–thaw cycles (each cycle: 24 h soaking, 24 h freezing at −27 °C, 24 h thawing)	30 wt.%	Polypropylene	23.1%	13.11%	28.17%	-	[81]

**Table 5:** Applications of bamboo fiber-reinforced polymer composites, aging conditions, and mitigation strategies.

Application Area	Common Aging Conditions	Effect on Product Performance	Mitigation Strategies
Automotive interior components	UV radiation, elevated temperatures, humidity fluctuations	Discoloration, cracking, delamination, and mechanical degradation	Use of UV stabilizing fillers, hybrid reinforcements
Automotive exterior panels	UV exposure, thermal cycling, rain/moisture,	Surface cracking and mechanical degradation	Use of UV stabilizing fillers, hybrid reinforcements
Construction panels	Moisture ingress, UV radiation, freeze–thaw cycles	Swelling, delamination	Surface treatment of fibers, use of UV stabilizing fillers, and hybrid reinforcements
Furniture and home decor	Direct sunlight (UV), ambient moisture	Microcracking and mechanical degradation	Use of fillers and hybrid reinforcements
Sporting goods	Moisture absorption and temperature variation	Moisture-induced delamination	Use of fillers and hybrid reinforcements
Building insulation panels	Hygrothermal aging	Physical and mechanical degradation	Surface treatment of fibers, use of fillers, and hybrid reinforcements

## Data Availability

No new data were created in this study.
